# Evaluation of the efficacy of prophylactic extended field irradiation in the concomitant chemoradiotherapy treatment of locally advanced cervical cancer, stage IIIB in the 2018 FIGO classification

**DOI:** 10.1186/s13014-019-1431-9

**Published:** 2019-12-16

**Authors:** Qingyu Meng, Xiaoliang Liu, Weiping Wang, Xiaorong Hou, Xin Lian, Shuai Sun, Junfang Yan, Zhikai Liu, Zheng Miao, Fuquan Zhang, Ke Hu

**Affiliations:** Department of radiation oncology, Peking Union Medical College Hospital, Chinese Academy of Medical Sciences and Peking Union Medical College, Beijing, People’s Republic of China

**Keywords:** FIGO IIIB cervical cancer, Extended field irradiation, Concomitant chemoradiotherapy, Acute toxicity

## Abstract

**Background:**

The new staging system of cervical cancer issued in 2018 by the International Federation of Gynecology and Obstetrics (FIGO), calls for a new evaluation of the efficacy of prophylactic extended field irradiation (EFI) in the concomitant chemoradiotherapy/brachytherapy treatment of locally advanced cervical cancer patients (stage IIIB).

**Methods:**

We performed a retrospective study consisting of 133 FIGO IIIB cervical cancer patients treated in the Peking Union Medical College Hospital from 2002 to 2010. The patients were distributed in two groups depending whether they were treated with EFI or pelvic only irradiation. The therapeutic efficacy, toxicity and prognostic factors of EFI were evaluated in the frame of the new FIGO staging system.

**Results:**

When compared to patients who received pelvic only irradiation, patients who received prophylactic EFI showed significantly less distant metastasis and a significant improvement in their 5 years overall survival (OS), disease free survival (DFS), out of field recurrence free survival (OFRFS) and para-aortic lymph node metastasis free survival (PALNMFS). Multivariate analysis revealed that EFI is an independent prognosis factor for DFS, OFRFS and PALNMFS. Finally, although more acute complications were observed in the EFI group, there is no significantly worst acute toxicity in the EFI group.

**Conclusion:**

Our retrospective analysis supports the prophylactic effect of EFI in the concomitant chemoradiotherapy treatment of IIIB patients and suggests that this prophylactic effect is associated with a clear improvement in 5-years OS, DFS, OFRFS and PALNMFS. Consequently, EFI appears to be a very valid treatment option for FIGO IIIB cervical cancer patients.

## Background

With about 570,000 new cases per year worldwide, cervical cancer is the fourth most frequent female cancer and causes about 300,000 deaths every year [1–3]. Cervical cancer usually progresses by steps, starting from regional pelvic lymph nodes followed by para-aortic lymph nodes (PALN) and finally by distant metastases with the predominance of PALN metastases [4, 5]. Recent advances in imaging technology together with the development of minimally invasive surgery contributed to the concerted efforts to decrease the mortality of cervical cancer and redefined the case management paradigm. Consequently, in 2018, the FIGO Gynecologic Oncology Committee has modified the cervical cancer staging system [6]. One of the main alterations is the classification of the stages IIIB and IIIC. The previous classification system does not explicitly consider lymph node metastasis [7]. In the new classification issued in 2018, the stage IIIB is characterized by the progression to the pelvic wall and/or hydronephrosis or nonfunctioning kidney without lymph node metastasis. Pelvic and/or abdominal aortic lymph node metastases are now promoted to stage IIIC.

Pelvic wall involvement is associated with an increased rate of retroperitoneal lymph node metastasis [8–10], which advocates for the use of prophylactic extended field exposure in the chemoradiotherapy treatment of IIIB patients. However, the new guidelines do not provide any specific direction concerning the need for prophylactic extended field exposure for IIIB patients. Prophylactic extended field irradiation (EFI) is commonly used for the treatment of occult PALN metastases in patients with advanced cervical cancer (former IIIB, now IIIC) [5, 11–13]. So far, reports on the efficiency of EFI are contradictory and do not provide clear guidance on the usage of EFI. For example, Rotman et al., have shown that EFI has no effect on locoregional tumor control, but is associated with an increased overall survival (OS) rate [11]. Whereas Haie et al. found that EFI had no effect on locoregional control or survival, but did lower the occurrence of PALN and remote metastases without pelvic failure [12].

These studies were done in the frame of the previous IIIB staging classification. In the light of the new classification, we preformed a retrospective study to analyze therapeutic efficacy, treatment failure, toxicity and prognostic factors of EFI for 133 FIGO IIIB cervical cancer patients treated by concomitant chemoradiotherapy in the Peking Union Medical College Hospital (PUMCH) from 2002 to 2010. We are able to show the efficacy of EFI in patients with locally advanced cervical cancer stage IIIB without PAN involvement.

## Methods

### Patient characteristics

In total, 133 FIGO IIIB cervical cancer patients were retrospectively reviewed in this study. The age of the patients ranged from 33 to 88 years old, with a median age of 50 years old. The clinical condition was determined by clinical checkup combined with biopsy analysis before the first treatment. Locally advanced cervical cancer was defined according to International Federation of Gynecology and Obstetrics (FIGO) staging as IIIB [6]. The patients were retrospectively assigned to two groups depending on whether or not they received prophylactic extended field irradiation. Patients’ details are summarized in Table 1.

**Table 1:** General patients’ information

Character	Group definition	Total	EFI	Pelvis Only	*P* value
Age	≥65	17	2	15	0.001
	< 65	116	65	51	
Pathology type	Squamous	122	63	59	0.332
	Adenocarcinoma, Adeno/squamous Carcinoma	11	4	7	
Tumor size	4 cm	33	18	15	0.581
	≤ 4 cm	100	49	51	
HGB prior treatment	< 110 g/L	36	22	14	0.144
	≥ 110 g/L	94	44	50	
Concurrent chemotherapy	≥ 4 cycles	94	50	44	0.313
	< 4 cycles	39	17	22	
SCC-Ag	< 10	55	31	24	0.657
	≥ 10	65	34	31	
EQD2 (point A)	< 90Gy	16	4	12	0.091
	90–98 Gy	32	18	14	
	≥ 98Gy	85	45	40	
Therapy duration	≤ 63 days	111	55	56	0.669
	> 63 days	22	12	10	

### Radiation therapy

All patients were scheduled to receive external beam radiation therapy and intracavity brachytherapy. The radiation therapies were administered as described elsewhere [14].

In our institution, patients who satisfied the following criteria received prophylactic extended field irradiation:

(1) No para-aortic LN metastasis (as monitored by CT, MRI or PET/CT)

(2) No evidence of distant metastasis;

(3) Pelvic wall involvement on both sides;

(4) ECOG score ranging from 0 to 2 points;

(5) Before treatment, levels of NEUT ≥1.5*10^9^.L^− 1^, HGB ≥ 80 g.L^− 1^, platelet ≥100 × 10^9^.L^− 1^, serum creatinine < 1.5 mg.dL^− 1^. AST and ALT are within 2 times of the upper limit of normality;

Exclusion criteria:

(1) Displays iliac lymph node metastasis;

(2) Have already undergone surgery for cervical cancer (including pelvic or retroperitoneal lymph node resection but excluding tumor biopsy), radiotherapy or chemotherapy;

(3) Has a history of malignant tumors;

(4) Already underwent abdominal or pelvic radiotherapy;

(5) Women during pregnancy or lactation;

(6) Present an active inflammatory bowel disease, or has a history of severe stomach and duodenal ulcer;

(7) Present an active infection, fever;

(8) Suffer from a serious disease such as unstable heart disease, kidney disease, chronic hepatitis, poorly controlled diabetes, and mental illness.

For the patients who received prophylactic extended field irradiation (forming the EFI group), the CTV covered the para-aortic lymph node regions additionally to the CTV of pelvic radiotherapy. Para-aortic regions encompassed the area adjacent to the aorta and inferior vena cava, with a lower border of the aortic bifurcation. The upper border of the extended field was usually at T12 or the renal vessel.

### Concurrent chemotherapy

Ninety-four patients received more than 4 cycles of concurrent chemotherapy, 39 patients were treated with less than 4 cycles. Out of these 39 patients, 17 patients didn’t receive any chemotherapy for personal reasons. Patients diagnosed as squamous were treated with a weekly cisplatin-based regimen at a dose of 40 mg/m2/week for 4 to 6 weeks. The adenocarcinoma patient received an additional PF regimen that consisted of cisplatin 70 mg/m2 on day 1 and fluorouracil 1000 mg/m2 from day 1 to day 4. The PF regimen was administered every 3 weeks for a total of 1–2 cycles. At the end of the treatment, the outcome was assessed following the guidelines proposed previously [15].

### Toxicity and adverse effect assessment

All patients were examined for toxicities and adverse effects every week during the treatment. The severity of acute complications was classified following the Common Terminology Criteria for Adverse Events (CTCAE v4.0) (https://ctep.cancer.gov/protocolDevelopment/ electronic_applications/docs/ctcv20_4–30-992.pdf). Late complications were graded following the RTOG/EORTC 1987 toxicity scales [16].

### Follow-up

Patients had a review check-up every 3 months during the first 2 years after the final treatment, and twice a year during the third to fifth year after treatment, and once a year starting from the fifth year after the last treatment. The review check-up includes blood biochemistry, SCC Ag, gynecological examination, pelvic MRI, chest and abdomen enhanced CT. The last follow up for the current study was carried out in November 2017.

### Statistics analysis

Overall survival (OS) is defined as the time from the start of treatment to the date of death or to the date of censoring. Disease-free survival (DFS) is defined as the time interval between the start of treatment and the detection of recurrence, metastasis or death. Local control rate (LCR) is defined as the percentage of the arrest of cancer growth at the site of origin. Out-of-field recurrence free survival (OFRFS) is defined as the beginning of radiotherapy to the detection of out of field recurrence or out of field recurrence related death. PALN metastasis free survival (PALNMFS) is defined as the beginning of radiotherapy to the detection of PALN metastasis or PALN metastasis related death OS, DFS, LCR, OFRFS and PALNMFS were calculated with the Kaplan-Meier method by using SPSS 17.0 statistical software and compared using the log-rank test. Log-rank method was also used to perform univariate analysis, when the factor was found significant (*P* < 0.05), the Cox regression model was used to run multivariate analysis. *P* value < 0.05 was considered statistically significant.

## Results

### Patient characteristics

The characteristics of the 133 patients are summarized in Table 1. The vast majority of the patients had squamous cell carcinomas (122 patients, 92%). 66 patients received pelvis only field irradiation, forming the pelvis only control group and 67 patients received prophylactic extended field irradiation and form the EFI test group. The only significant difference between the two groups is the distribution of patients around the 65-year-old threshold: 2 patients are older than 65 in the EFI group against 15 in the pelvis only group.

### Treatment outcome evaluation

After treatment, we observed 19 cases of local recurrence with no significant difference between the EFI (8 cases) and Pelvis only group (11 cases), 27 patients with out-of-field recurrence including 6 patients with PALN metastasis (Additional file 1: Table S1). Interestingly, there are significantly less patients with out-of-field recurrence in the EFI group (7 patients) than in the Pelvis only group (20 patients) (*p* = 0,004). In particular, no metastasis of the retroperitoneal lymph node was observed in the EFI group against 6 for the pelvic only group (*p* = 0,011), confirming the prophylactic efficiency of EFI.

The 5-year OS, DFS, LCR, OFRFS were 73.3, 68.9, 85.5 and 81.6% (Additional file 3: Figure S1A-D), suggesting the standard concurrent chemoradiotherapy is indeed very effective for FIGO IIIB cervical cancer patients classified following the new staging parameters.

**Fig. 1:**
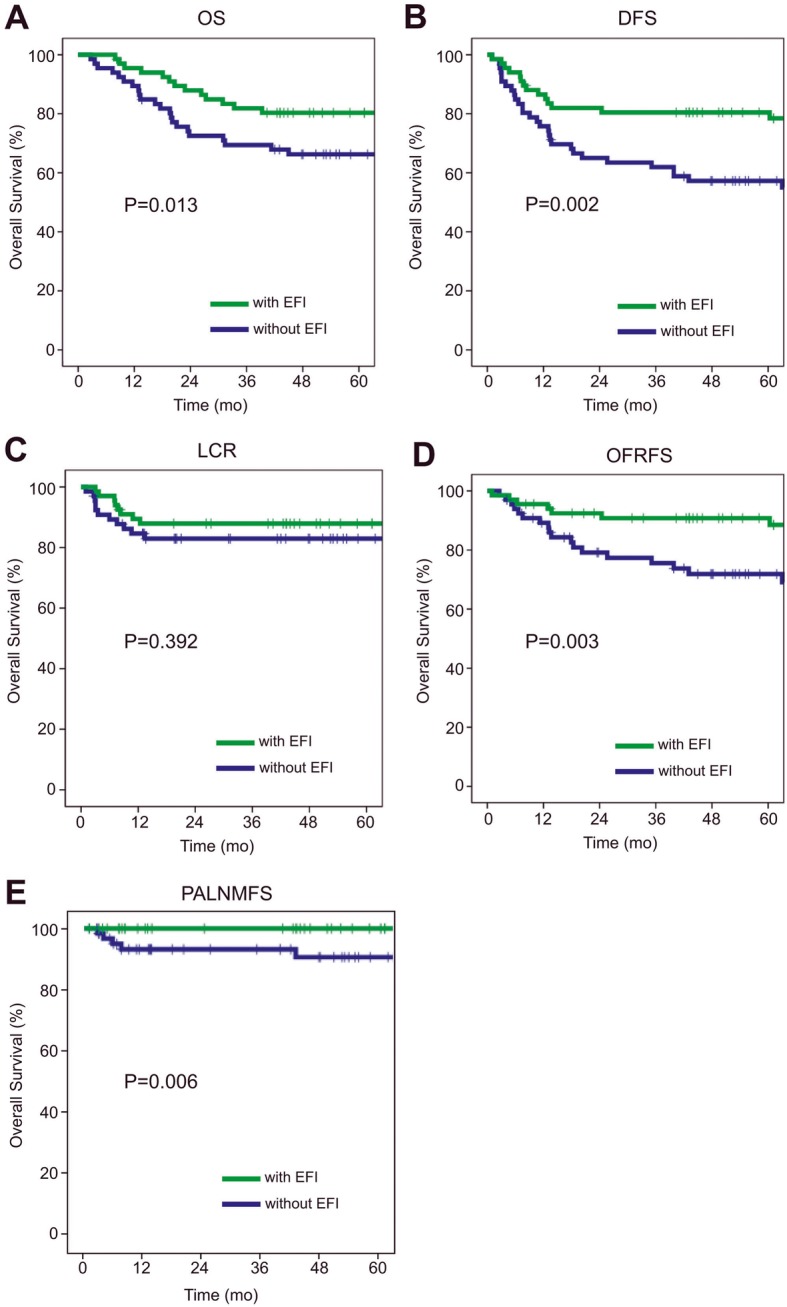
Comparison of the OS, DFS, LC, OFRFS and PLANMFS between the EFI and Pelvic only groups (green and blue traces respectively)

When comparing the 5 years OS, DFS, LCR, OFRFS and PALNMFS between the EFI and pelvis only groups (Fig. 1a-e), significant differences are seen in the OS, DFS, OFRFS and PALNMFS. The OS has dramatically improved from 66.3% for the pelvic only group to 80.3% for the EFI group (*p* = 0,013). The DFS from 57,2 to 80,4 (*p* = 0,002). The OFRFS rose from 71,9% for the pelvic only group to 90,8% for the EFI group (*p* = 0,003) and the PALNMFS rose from 90,8 to 100% (*p* = 0.006). However, there is not significant difference in the 5 years LCR between the two groups (Fig. 1c). Taken together, these data suggest that EFI indeed carries a prophylactic effect for IIIB patients (no retroperitoneal lymph node metastasis observed in the EFI group against 6 in the pelvic only group) and that this prophylactic effect is associated with a clear improvement in OS, DFS and OFRFS.

### Treatment toxicity

We also have carefully evaluated the acute and delayed toxicity associated with the treatment (Table 2).

**Table 2:** Acute and delayed toxicity after treatment

	*EFI*				*Pelvis only*				
*Grade*	*II*	*III*	*IV*	*Total*	*II*	*III*	*IV*	*Total*	*P value*
*Acute toxicity (CTCEA 2.0)*									
*Leukocytes*	*18*	*35*	*4*	*57*	*18*	*23*	*0*	*41*	*0,003*
*Neutrophils*	*28*	*16*	*2*	*46*	*16*	*10*	*3*	*29*	*0,004*
*Platelets*	*17*	*8*	*0*	*25*	*11*	*6*	*0*	*17*	*0,152*
*Lymphopenia*	*2*	*61*	*0*	*63*	*23*	*39*	*0*	*62*	*0,982*
*Hemoglobin*	*31*	*16*	*3*	*50*	*30*	*4*	*2*	*36*	*0.015*
*Frequent urination*	*28*	*2*	*0*	*30*	*28*	*2*	*0*	*30*	*0,461*
*Diarrhea*	*12*	*7*	*0*	*19*	*18*	*4*	*0*	*22*	*0,332*
*Delayed toxicity (RTOG/EORTC1987)*									
*Urinary system*	*5*	*3*	*0*	*8*	*3*	*1*	*0*	*4*	*0.190*
*Lower digestive tract*	*6*	*1*	*0*	*7*	*4*	*1*	*0*	*5*	*0,392*

Only a few cases of delayed toxicity were observed and no significant difference was observed between the two groups for delayed toxicity.

However, significant differences in acute toxicity were observed between the two groups. Compared to the Pelvic only group, the EFI group contained significantly more patients suffering from low counts of leukocyte, neutrophils and low level of hemoglobin.

### Prognostic factors analysis

In order to identify FIGO IIIB cervical cancer prognosis factors, the 133 patients were distributed according to the difference in their treatment (Additional file 2: Table S2). A univariate analysis was performed in order to identify the prognostic factors for OS, DFS, LCR, OFRFS and PALNMFS. Prognostic factors found to be significant (*P* < 0.05) by univariate analysis were then further analyzed using multivariate analysis (Table 3). Our results suggested that age, tumor size and EQD2 were independent prognostic factors for OS; age, EQD2 and EFI were independent prognostic factors for DFS; the independent prognostic factors for LCR included tumor size, age and EQD2; concurrent chemotherapy cycles and EFI were independent prognostic factors for OFRFS and EFI was independent prognostic factor for PALNMFS.

**Table 3:** Multivariate analysis for prognostic factors

*Subject*	*HR*	*CI 95%*	*P value*
*OS*			
*Age*	0.331	0.160–0.683	0.003
*Tumor size*	0.262	0.098–0.699	0.007
*EQD2(point A)*	2.530	1.159–5.521	0.036
*DFS*			
*Age*	0.462	0.232–0.920	0.028
*EFI*	1.927	1.000–3.714	0.050
*EQD2(point A)*	2.949	1.379–6.306	0.005
*LCR*			
*Tumor size*	0.091	0.011–0.745	0.026
*Age*	0.266	0.084–0.843	0.024
*EQD2(point A)*	5.578	1.795–17.332	0.003
*OFRFS*			
*Concurrent chemotherapy*	2.738	1.248–6.009	0.012
*EFI*	3.287	1.369–7.892	0.008

## Discussion

Concurrent chemoradiotherapy (CCRT) remains the principal treatment option for patients with locally advanced cervical cancer [17–20]. Locally advanced carcinoma is often associated with occult para-aortic metastases [12, 21–28], which advocates for the use of the prophylactic extended field irradiation (EFI) rather than pelvic only irradiation. However, since its implementation, reports on the efficacy of prophylactic EFI in CCRT have been contradictory. Rotman et al. report an improved overall 10 years survival for the patients who received EFI but didn’t observe any difference in disease free survival [11]. Meng et al. observed a significant improvement in disease free survival for patients treated with EFI as well as a trend towards higher OS and DMFS [29]. On the other hand, Haie et al. observed that EFI only reduced the rates of PALN and distant metastases without affecting survival [12]. Additionally, EFI seems to be associated with elevated toxicity and higher risks of side effects. Haie et al. reported an increase in severe digestive complications for patients treated with EFI [12] while Rotman et al reported an higher cumulative incidence of grade 4 and 5 toxicities at 10 years for patients who received EFI as well as a higher (although not significant) death rate due to radiotherapy complications [11].

In the new classification issued in 2018, the stage IIIB is characterized by the extension to the pelvic wall and/or hydronephrosis or nonfunctioning kidney. Since pelvic wall involvement correlates with the increase of the rate of retroperitoneal lymph node metastasis [8–10], the efficiency of prophylactic EFI becomes particularly relevant. However, the new guidelines do not provide any specific direction concerning the use of prophylactic EFI for IIIB patients. This lack of direction calls for a reconsideration of the effects of EFI in the CCRT treatment of locally advanced cervical cancer (FIGO IIIB). Indeed, since the release of the new classification, the effects and toxicity of EFI on FIGO IIIB cervical cancer patients have not been examined.

In this study we present a retrospective analysis of the therapeutic efficacy, treatment failure, toxicity and prognostic factors of EFI for 133 FIGO IIIB cervical cancer patients treated by concomitant chemoradiotherapy in the Peking Union Medical College Hospital (PUMCH) from 2002 to 2010.

Interestingly, when compared to patients who received pelvic only irradiation (66 cases), patients who received prophylactic extended field irradiation (67 patients) showed significantly less out-of-field metastases and no retroperitoneal lymph node metastasis at all. In addition patients treated with EFI exhibited clear improvement in their 5 years OS, DFS, OFRFS and PALNMFS (Fig. 1). However, no significant difference was found for the 5 years LCR. Along this line, the multivariate analysis revealed that EFI is an independent prognosis factor for DFS and DMFS.

As already mentioned, toxicity is an important concern for patients treated with EFI. Our study showed that patients experiencing EFI are more likely to suffer from blood toxicities (leukocyte, neutrophils and hemoglobin). However, no significant differences were observed between the two groups in terms of delayed toxicity.

Alongside treatment toxicity, lumbar vertebral compression fractures after radiotherapy are often reported in the literature [13]. However, in our study, no case of lumbar vertebral compression fracture caused by radiotherapy has been observed. This may be due to the fact that we used conformal or intensity-modulated radiotherapy techniques. It is well known that intensity-modulated radiation therapy has an excellent dose distribution and conformability. Additionally, the feasibility of each treatment plan was carefully evaluated, ensuring that the dose is evenly distributed and avoiding any hotspots falling on important radioactive-sensitive organs.

Finally, patient’s quality of life (QOL) is also an important criterion to assess a treatment. Unfortunately, due to the retrospective nature of the study no quantitative measurement of the patient’s QOL is available. However, the trend was that no noticeable difference in patient’s QOL was observed between the two groups.

All together, our retrospective analysis supports the prophylactic effect of EFI in the chemoradiotherapy treatment of IIIB patients and seems to suggest that this prophylactic effect is associated with a clear improvement in OS, DFS OFRFS and PALNMFS. Consequently, EFI appears to be a very effective treatment option for IIIB cervical cancer patients in the new FIGO classification. Interestingly, our results differ from our analysis based on the previous staging standards [29], which demonstrates the importance of revaluating the effects of EFI in the frame of the new FIGO classification.

Unavoidably, our study conveys limitations. First, this is a single-center center study with a limited number of patients. In a multi-center study with a larger sample size, some trends we observed could be confirmed. Additionally, the discrepancy that EFI is associated with higher 5 years OS but doesn’t not appeared to be a prognosis factor for OS could also be solved with a larger sample size. Second, this is a retrospective study. As a consequence, patients were not perfectly matched in the two groups. More studies will need to be conducted in order to reach a consensual view on the effect of EFI for the new FIGO stage IIIB cervical cancer patients.

## Conclusion

Our retrospective study supports the prophylactic effect of EFI in the chemoradiotherapy treatment of IIIB patients and that this prophylactic effect is associated with a clear improvement in 5-years OS, DFS, OFRFS and PALNMFS. We anticipate that our observations will help guiding the use of prophylactic EFI in the treatment of stage IIIB cervical cancer patients.

## Supplementary information

**Additional file 1: Table S1.** Details for treatment failure patterns.

**Additional file 2: Table S2.** Univariate analysis for prognostic factors.

**Additional file 3: Figure S1.** OS, DFS, LC and OFRFS of the complete patient’s cohort.

## Data Availability

The datasets used and/or analyzed for the present study are available from the corresponding author on reasonable request.

## Abbreviations

CCRT: Concomitant chemoradiotherapy
CT: Computer tomography
CTV: Clinical tumor volume
DFS: Disease free survival
DMFS: Distant metastasis free survival
EFI: Extended field irradiation
EORTC: European Organization for Research and Treatment of Cancer
EQD: Equivalent dose
FIGO: Federation of gynecology and obstetrics
LCR: Local control rate
LN: Lymph node
OFRFS: Out-of-field recurrence free survival
OS: Overall survival
PALN: Para aortic lymph node
PALMFS: PALN metastasis free survival
PET: Positron emission tomography
RTOG: Radiation Therapy Oncology Group
SCC-Ag: Squamous cell carcinoma antigen
