# Supplementary material for: Evaluation of the efficacy of prophylactic extended field irradiation in the concomitant chemoradiotherapy treatment of locally advanced cervical cancer, stage IIIB in the 2018 FIGO classification
Source: Radiat Oncol. 2019 Dec 16;14:228. doi: 10.1186/s13014-019-1431-9 (PMC6915883; doi:10.1186/s13014-019-1431-9)
Supplement: Supplementary file 1 — Additional file 1: Table S1. Details for treatment failure patterns. [file 13014_2019_1431_MOESM1_ESM.docx]

**Table S1. Details for treatment failure patterns.**

|  | Total | EFI | Pelvis only | P Value |
| --- | --- | --- | --- | --- |
| Local recurrence | 19 | 8 | 11 | 0,0397 |
| Local progression | 8 | 1 | 6 | 0,139 |
| Cervical recurrence | 3 | 1 | 2 | 0,55 |
| Parametrial recurrence | 3 | 1 | 2 | 0,55 |
| Vaginal recurrence | 3 | 2 | 1 | 0,568 |
| Uterus recurrence | 1 | 1 | 0 | 0,319 |
| Vulva metastasis | 1 | 0 | 1 | 0,312 |
| Pelvic recurrence | 1 | 1 | 0 | 0,319 |
| Out of field recurrence | 27 | 7 | 20 | 0,004 |
| Lung | 13 | 4 | 9 | 0,129 |
| Liver | 3 | 1 | 2 | 0,55 |
| Bone | 4 | 1 | 3 | 0,303 |
| Adrenal | 1 | 0 | 1 | 0,312 |
| Supraclavicular lymph node | 1 | 1 | 0 | 0,319 |
| Mediastinal lymph node | 2 | 1 | 1 | 0,991 |
| Retroperitoneal lymph node | 6 | 0 | 6 | 0,011 |
|  |  |  |  |  |

Note: some patients showed multiple subtypes of local recurrence and metastasis in different organs.
