# Supplementary material for: Evaluation of the efficacy of prophylactic extended field irradiation in the concomitant chemoradiotherapy treatment of locally advanced cervical cancer, stage IIIB in the 2018 FIGO classification
Source: Radiat Oncol. 2019 Dec 16;14:228. doi: 10.1186/s13014-019-1431-9 (PMC6915883; doi:10.1186/s13014-019-1431-9)
Supplement: Supplementary file 2 — Additional file 2: Table S2. Univariate analysis for prognostic factors. [file 13014_2019_1431_MOESM2_ESM.docx]

Table S2. Univariate analysis for prognostic factors.

| *Subject Classification (n)* | | *5 years’ survival (%)* | | | | | |
| --- | --- | --- | --- | --- | --- | --- | --- |
|  |  | OS | DFS | LCR | OFRFS | PALNMFS |  |
| *Age* | ≥65(17) | 64.7% | 52.9% | 70.6% | 81.9% | 92.9% |  |
|  | <65(116) | 74.6% | 71.3% | 87.7% | 81.5% | 96.0% |  |
|  |  | p=0.005 | p=0.001 | p=0.045 | p=0.584 | p=0.622 |  |
| *Histological type* | Squamous (122) | 75.1% | 71.1% | 85.9% | 83.5% | 95.3% |  |
|  | Adenocarcinoma, Adeno/squamous  Carcinoma (11) | 53.0% | 45.5% | 81.8% | 60.6% | 100.0% |  |
|  |  | p=0.218 | p=0.286 | p=0.772 | p=0.224 | p=0.463 |  |
| *Tumor size* | ＜4cm(33) | 90.9% | 87.9% | 97.0% | 90.9% | 93.6% |  |
|  | ≥4cm (100) | 67.4% | 62.6% | 81.6% | 78.0% | 96.3% |  |
|  |  | p=0.013 | p=0.101 | p=0.035 | p=0.327 | p=0.216 |  |
| *HGB level* | ＜110 g/L(36) | 63.8% | 61.1% | 83.0% | 70.9% | 100.0% |  |
|  | ≥110 g/L (94) | 77.2% | 73.1% | 87.1% | 86.3% | 95.1% |  |
|  |  | p=0.238 | p=0.501 | p=0.548 | p=0.150 | p=0.193 |  |
| *Concurrent chemotherapy* | ≥4 cycles (94) | 76.3% | 71.1% | 85.0% | 86.0% | 97.3% |  |
|  | <4 cycles (39) | 66.0% | 63.6% | 86.9% | 70.8% | 91.5% |  |
|  |  | p=0.024 | p=0.078 | p=0.887 | p=0.019 | p=0.225 |  |
| *SCC-Ag* | ＜10(55) | 83.6% | 80.0% | 89.1% | 90.2% | 100.0% |  |
|  | ≥10(65) | 68.4% | 65.6% | 87.5% | 74.2% | 92.6% |  |
|  |  | p=0.182 | p=0.099 | p=0.800 | p=0.030 | p=0.028 |  |
| *EQD2*  *(point A）* | <90Gy (16) | 49.2% | 37.5% | 55.6% | 66.7% | 90.9% |  |
|  | 90-98 Gy (32) | 68.0% | 68.6% | 80.8% | 86.9% | 96.7% |  |
|  | ≥98Gy (85) | 79.8% | 75.1% | 92.8% | 82.6% | 96.0% |  |
|  |  | p=0.002 | p=0.001 | p=0.000 | p=0.168 | p=0.422 |  |
| *Therapy*  *duration* | ≤63 days (111) | 76.3% | 72.8% | 85.4% | 84.5% | 96.8% |  |
|  | >63 days (22) | 57.1% | 48.5% | 85.9% | 66.5% | 89.7% |  |
|  |  | p=0.092 | p=0.057 | p=0.924 | p=0.053 | p=0.202 |  |
| *EFI* | w/o（66） | 66.3% | 57.2% | 82.9% | 71.9% | **90.8%** |  |
|  | w/t（67） | 80.3% | 80.4% | 87.9% | 90.8% | **100.0%** |  |
|  |  | p=0.013 | p=0.002 | p=0.392 | p=0.003 | **p=0.006** |  |
