# Supplementary figures and images for: Evaluation of the efficacy of prophylactic extended field irradiation in the concomitant chemoradiotherapy treatment of locally advanced cervical cancer, stage IIIB in the 2018 FIGO classification
Source: Radiat Oncol. 2019 Dec 16;14:228. doi: 10.1186/s13014-019-1431-9 (PMC6915883; doi:10.1186/s13014-019-1431-9)

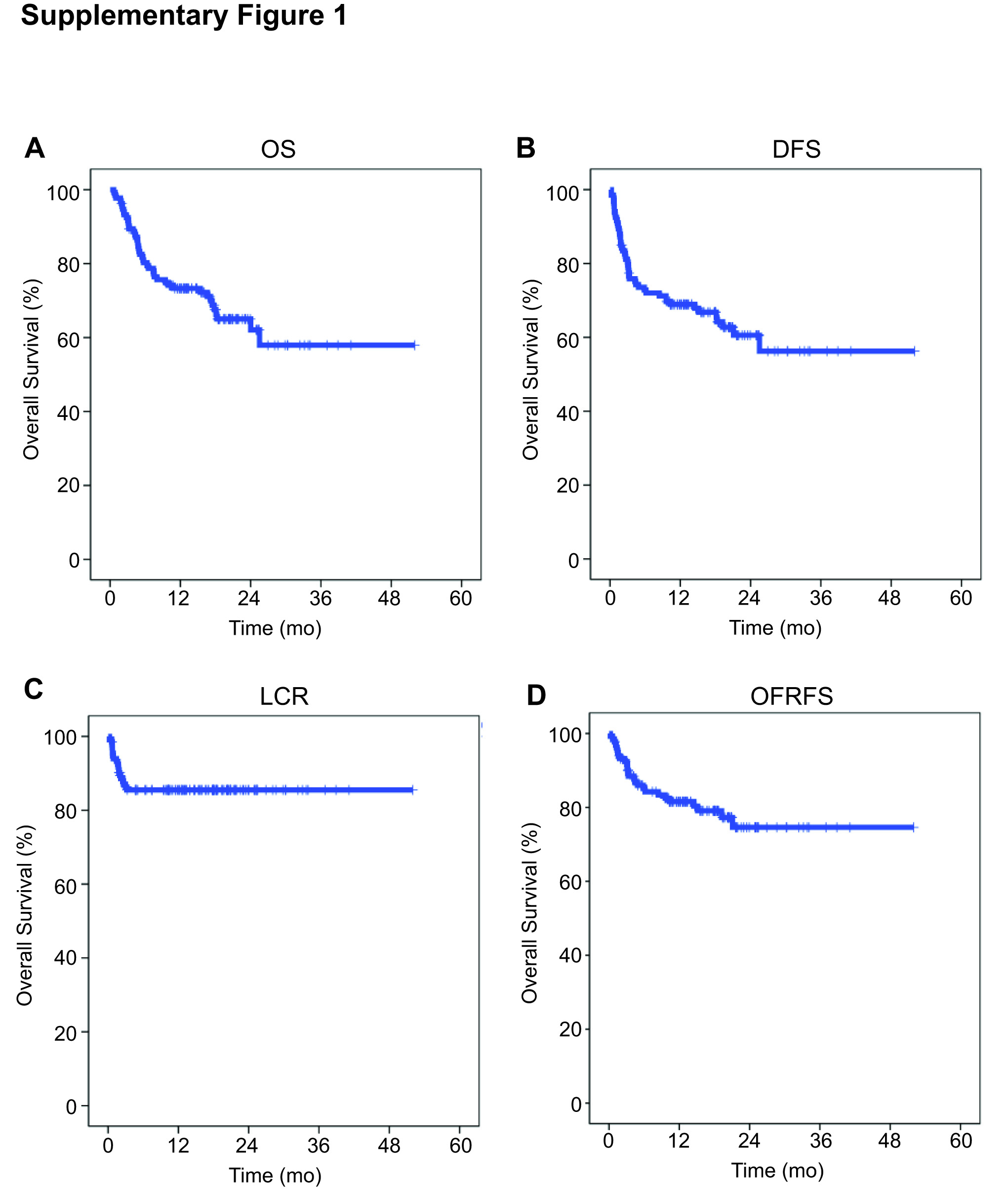

Supplement: Supplementary file 3 — Additional file 3: Figure S1. OS, DFS, LC and OFRFS of the complete patient’s cohort. [file 13014_2019_1431_MOESM3_ESM.jpg]
